# Differentiation of angiogenic burden in human cancer xenografts using a perfusion-type optical contrast agent (SIDAG)

**DOI:** 10.1186/bcr1875

**Published:** 2008-03-10

**Authors:** Alexander Wall, Thorsten Persigehl, Peter Hauff, Kai Licha, Michael Schirner, Silke Müller, Angelika von Wallbrunn, Lars Matuszewski, Walter Heindel, Christoph Bremer

**Affiliations:** 1 Department of Clinical Radiology, University Hospital of Münster, Albert-Schweitzer-Straße 33, 48129 Münster, Germany; 2 Global Drug Discovery, Bayer Schering Pharma AG, Müllerstraße 178, 13353 Berlin, Germany; 3 Institute of Veterinary Pathology, Freie Universität Berlin, Robert-von-Ostertag-Sraße 15, 14163 Berlin, Germany; 4 Interdisciplinary Center for Clinical Research (IZKF Muenster, FG3), University of Münster, Domagkstraße 3, 48149 Münster, Germany

## Abstract

**Introduction:**

Use of fluorescence imaging in oncology is evolving rapidly, and nontargeted fluorochromes are currently being investigated for clinical application. Here, we investigated whether the degree of tumour angiogenesis can be assessed *in vivo *by planar and tomographic methods using the perfusion-type cyanine dye SIDAG (1,1'-bis- [4-sulfobutyl]indotricarbocyanine-5,5'-dicarboxylic acid diglucamide monosodium).

**Method:**

Mice were xenografted with moderately (MCF7, DU4475) or highly vascularized (HT1080, MDA-MB435) tumours and scanned up to 24 hours after intravenous SIDAG injection using fluorescence reflectance imaging. Contrast-to-noise ratio was calculated for all tumours, and fluorochrome accumulation was quantified using fluorescence-mediated tomography. The vascular volume fraction of the xenografts, serving as a surrogate marker for angiogenesis, was measured using magnetic resonance imaging, and blood vessel profile (BVP) density and vascular endothelial growth factor expression were determined.

**Results:**

SIDAG accumulation correlated well with angiogenic burden, with maximum contrast to noise ratio for MDA-MB435 (*P *< 0.0001), followed by HT1080, MCF7 and DU4475 tumours. Fluorescence-mediated tomography revealed 4.6-fold higher fluorochrome concentrations in MDA-MB435 than in DU4475 tumours (229 ± 90 nmol/l versus 49 ± 22 nmol/l; *P *< 0.05). The vascular volume fraction was 4.5-fold (3.58 ± 0.9% versus 0.8 ± 0.53%; *P *< 0.01), blood vessel profile density 5-fold (399 ± 36 BVPs/mm^2 ^versus 78 ± 16 BVPs/mm^2^) and vascular endothelial growth factor expression 4-fold higher for MDA-MB435 than for DU4475 tumours.

**Conclusion:**

Our data suggest that perfusion-type cyanine dyes allow assessment of angiogenesis *in vivo *using planar or tomographic imaging technology. They may thus facilitate characterization of solid tumours.

## Introduction

Contrast-enhanced optical imaging is an emerging modality that may be used to detect and characterize solid tumours such as breast cancer [1,2]. Optical techniques based on intrinsic optical contrast (such as diffuse optical tomography) can provide spectroscopic information about physiological and functional tissue parameters (for instance, tissue oxygenation) [3,4]. However, there have been several unsuccessful attempts to demonstrate that nonenhanced optical mammography has diagnostic utility [5]. More recently, substantial effort has been invested in the development of fluorescent probes, which potentially can increase cancer to noncancer tissue contrast and therefore improve sensitivity and specificity of breast cancer imaging [4,6]. Indeed, indocyanine green (ICG)-enhanced diffuse optical mammography was successfully applied to detection of breast lesions in a proof-of-concept study [7]. However, factors such as rapid uptake by liver tissue, small quantum yield and low stability in watery solutions render ICG unsuitable as an optical contrast agent for detection of breast tumours [8].

**Figure 1:**
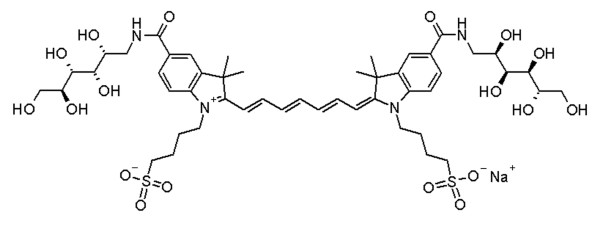
SIDAG. Presented is the chemical structure of SIDAG (1,1'-bis- [4-sulfobutyl]indotricarbocyanine-5,5'-dicarboxylic acid diglucamide monosodium).

Hence, other cyanine-based contrast agents have recently been developed that possess more favourable optical and pharmacokinetic properties. SIDAG (1,1'-bis- [4-sulfobutyl]indotricarbocyanine-5,5'-dicarboxylic acid diglucamide monosodium; Global Drug Discovery, Bayer Schering Pharma AG, Berlin, Germany) is among these recently synthesized derivatives of ICG that have improved photophysical and pharmacological characteristics (Figure 1) [8,9]. Based on recent developments in optical imaging, it is conceivable that the first clinically applied fluorochromes will be perfusion-type (nontargeted) contrast agents. However, it is currently unclear whether nontargeted, low-molecular-weight fluorochromes that are currently on the verge of being clinically introduced will allow us to extrapolate surrogate markers of tumour angiogenesis using planar or tomographic optical imaging. The aim of the present study was to explore the potential of SIDAG in differentiating the angiogenic burden associated with various cancer xenografts.

## Materials and methods

### Fluorochrome

The synthesis and physicochemical characteristics of SIDAG are described in detail elsewhere [9-11]. Compared with ICG, SIDAG exhibits improved hydrophilicity and lower plasma protein binding, with a free fraction of the fluorochrome of approximately 40% (ICG has 95% to 100% plasma protein binding). The excitation peak of the fluorochrome is 753 nm in phosphate-buffered saline and 755 nm in plasma, while the emission peak is around 790 nm for both media. A detailed description of the properties of SIDAG can be found elsewhere [9-12].

### Cell culture and tumour models

All primary cell lines (HT1080 fibrosarcoma, MCF7 breast adenocarcinoma, MDA-MB435 melanoma and DU4475 adenocarcinoma) used in this study were obtained from the American Tissue Culture Collection (Manassas, VA, USA). All cell lines were cultured in modified Eagle's medium, RPMI-1640, or Dulbecco's modified Eagle's medium cell media supplemented with 10% foetal calf serum and 1% penicillin/streptomycin (all products from Invitrogen Corporation, San Diego, CA, USA). Cells were grown using routine procedures in a monolayer culture at 37°C in a 5% carbon dioxide humidified atmosphere. To grow and propagate the tumours in mice, 2 × 10^6 ^cells were injected into the mouse flank (*nu*/*nu*; Charles Rivers, Sulzfeld, Germany). In the preliminary set of experiments at the beginning of the project, the pharmacokinetic properties of the fluorochrome and the optimal time window for animal scanning had to be determined. For this purpose, four animals bearing MDA-MB435 tumours were scanned repetitively up to 72 hours after probe injection (30 minutes, and 1, 2, 3, 6, 12, 24, 48 and 72 hours). Based on these data, all other animals were scanned 3, 6 and 24 hours after injection. Because MCF7 cells are hormone dependent, an oestradiol pellet (0.72 mg/pellet; Innovative Research of America, Sarasota, USA) was implanted subcutaneously 1 week before tumour inoculation. All tumours were allowed to grow to approximately 5 to 7 mm in diameter before imaging studies were performed. In order to assess whether fluorochrome accumulation in different xenografts can reliably be distinguished even in the same individuals, five animals were co-implanted with both MDA-MB435 and DU4475 tumours. A total of 43 tumours were examined and evaluated in this study.

### *In vivo *optical imaging studies

For reflectance and tomographic imaging studies, the mice were anaesthetized by intraperitoneal administration of ketamine (125 mg/kg body weight) and xylazine (12.5 mg/kg body weight). The average time of anesthesia was approximately 20 to 30 minutes and animals were allowed to wake up between imaging sessions. Before injection of the contrast agent, an anatomical white light image was obtained. SIDAG was dissolved in saline to achieve a 0.2 mmol/l solution and injected into the tail vein at a dose of 2 μmol/kg body weight. All experiments were performed with approval of the Institutional Animal Care Committee.

### Fluorescence reflectance imaging

The whole body multichannel small animal imager is a prototype reflectance imager and has been described elsewhere [13] (BonSAI – prototype imager; Siemens Medical Solutions, Erlangen, Germany). The acquisition time for near-infrared fluorescence images was 1 second at maximum photon flux of the system. Images were acquired using a charged-coupled device camera sensitive to near infrared light (Hamamatsu Photonics, Hamamatsu – City, Japan) and processed on a PC-based system. For signal quantification, the contrast-to-noise ratio was determined by region of interest (ROI) analysis (about 900 pixels) in the region of the tumour. ROIs of the same size were placed in nontarget tissue (hip muscle) and in the background beside the mouse. The contrast-to-noise ratio (CNR) was calculated as CNR = (SI_tumour _– SI_muscle_)/SD_noise _(where SI_tumour _is the signal intensity of the tumour, SI_muscle _is the signal intensity of the muscle and SD_noise _is the standard deviation of background).

### Fluorescence mediated tomography

All tomographic optical imaging studies were performed by a small-animal fluorescence mediated tomography (FMT) system (VisEn FMT™; VisEn Medical Inc., Woburn, MA, USA), which has been described in detail elsewhere [14]. FMT imaging was conducted 24 hours after injection of SIDAG immediately after the last fluorescence reflectance imaging (FRI) scan in a subset of animals (n = 4 for MDA-MB435 and DU4475, n = 5 for HT1080 and n = 6 for MCF7). Animal scan times ranged from 2 to 5 minutes; tomographic reconstruction times were approximately 1 to 3 minutes. The fluorochrome concentration inside the tumour tissue was measured using volume of interest analysis. Briefly, images were displayed as raw and as reconstructed three-dimensional datasets in transverse, sagittal and coronal planes. The target volume was defined by ROI placement in all three reconstructed planes.

Fluorochrome concentration in the target tissue was then automatically calculated from the reconstructed images.

### Magnetic resonance imaging and determination of vascular volume fraction

Magnetic resonance imaging (MRI) was performed using a clinical 3 Tesla magnetic resonance system (Intera; Philips Medical Systems, Best, The Netherlands) using a custom-made solenoid coil for animal imaging with a diameter of 70 mm (Philips Medical Systems, Philips, Hamburg, Germany). Tumour-bearing animals (n = 4 for each xenograft) were chosen randomly from the each group and were anaesthetized by intraperitoneal injection of ketamine (125 mg/kg body weight) and xylazine (12.5 mg/kg body weight). For stable vascular access, a jugular vein catheter (SIMS Portex, Kent, UK) was inserted [15]. Vascular volume fraction (VVF) measurements were performed as described elsewhere [16]. In our study the magnetic resonance relaxation time T2* and the R2* relaxation rate (1/T2*), respectively, were measured before and after intravenous injection of ultrasmall superparamagnetic iron oxide crystals (80 μmol Fe/kg body weight; SHU 555 C; Bayer Schering Pharma AG, Berlin, Germany) using a multiecho sequence with the following parameters: repetition time 474 ms, echo time 32 ms, Δecho time 5.8 ms, 11 echoes, slice thickness 1 mm, field of view 50 × 50 mm, matrix 192 × 192 (reconstructed 256 × 256), voxel size 0.2 × 0.2 × 1.0 mm, and total scan duration 75 seconds for four slices. ΔR2* maps were calculated and quantitative ΔR2* data were measured in ROIs covering the whole tumour volume. The VVF was calculated by calibrating the ΔR2* of the tumour with the corresponding ΔR2* of muscle tissue, which has an average VVF of 1.89% [17].

### Immunhistochemistry and calculation of blood vessel profile density

Six samples from each tumour type were chosen randomly and prepared, and each was embedded and snap frozen at -70°C immediately after imaging. For each tumour sample, on average 12 sections were selected using a systematic random sampling principle, as was described by Gundersen and Jensen [18]. Immunohistochemistry was performed on these cryostat sections (5 μm) using a biotynilated monoclonal rat-anti-mouse CD31 antibody (Pecam-1, MEC; BD Biosciences Pharmingen, San Jose, CA, USA) and the streptavidin-biotin-peroxidase technique. The blood vessel profile density (BVPD) was analyzed using a microscope (Axioskop 40; Zeiss, Göttingen, Germany) equipped with a mechanical stage linked to a computer running a stereological program (StereoInvestigator; MicroBrightField Inc., Williston, VT, USA). A camera (DXC-950P, 3CCD color video camera; Sony, Atsugi, Japan) connected to the microscope was used to transfer the microscopic picture to the computer screen. After defining the ROI and the number of desired fields of view at a magnification of 53×, the computer program defines and moves automatically to the analysis fields and randomly projects onto them an unbiased counting frame for blood vessel profile counting at a magnification of 530×. The counting rule applied to the counting tool and the evaluation of the number of profiles per area (in this case the blood vessel profile density [BVPD]) were in accordance with the method described by Gundersen and coworkers [18]. The BVPD is expressed as blood vessel profiles (BVPs)/mm^2 ^(BVP/mm^2^).

### Western blotting

Secreted vascular endothelial growth factor (VEGF) was analyzed in the supernatant of cultivated tumour cells that had been maintained in serum-free medium for 24 to 48 hours [19]. The supernatant from each cell line (equivalent to 1 × 10^7 ^cells) was collected and purified by centrifugation (1,500 *g *at 4°C for 10 minutes and 10,000 *g *at 4°C for 30 minutes). To precipitate and thus concentrate the protein, a twofold volume of cold acetone was added to each sample and maintained at -20°C for 16 hours, followed by centrifugation at 10,000 *g *at 4°C for 30 minutes. The protein pellets were dissolved in 100 μl of 6× SDS sample buffer (New England Biolabs, Beverley, MA, USA). Proteins were then electrophoretically separated in nonreducing 12% SDS-PAGE and transferred to polyvinylidene fluoride membranes (Millipore Corporation, Bedford, MA, USA). After blocking, immunoblots were incubated with VEGF polyclonal antibody (VEGF A-20: *sc-152*; Santa Cruz Biotechnology, Santa Cruz, CA, USA) and then with a peroxidase-conjugated goat anti-rabbit IgG secondary antibody (Sigma, St. Louis, MO, USA). Peroxidase activity was revealed using ECL chemiluminescence (Amersham Bioschiences, Piscataway, NJ, USA) and X-ray film, as described by the manufacturers. Recombinant human VEGF_165 _(Oncogene, Cambridge, MA, USA) was used as a positive control. The developed films were scanned in an AlphaImager 2200 (Alpha Innotech Corp., San Leandro, CA, USA) and the amount of protein was quantified using the provided Alpha Ease FC software (version 3.2.1). For comparison, the VEGF protein expression level in MDA-MB435 cells was set at 100%, and all other cell types were scaled accordingly.

### Statistical analysis

Data are presented as mean ± standard deviation. Differences between the tumour xenografts (CNR, SIDAG concentration and VVF) were statistically evaluated by analysis of variance with Bonferroni correction for multiple comparisons. *P *< 0.05 was regarded to indicate statistically significant differences.

## Results

### Near-infrared reflectance imaging

**Figure 2:**
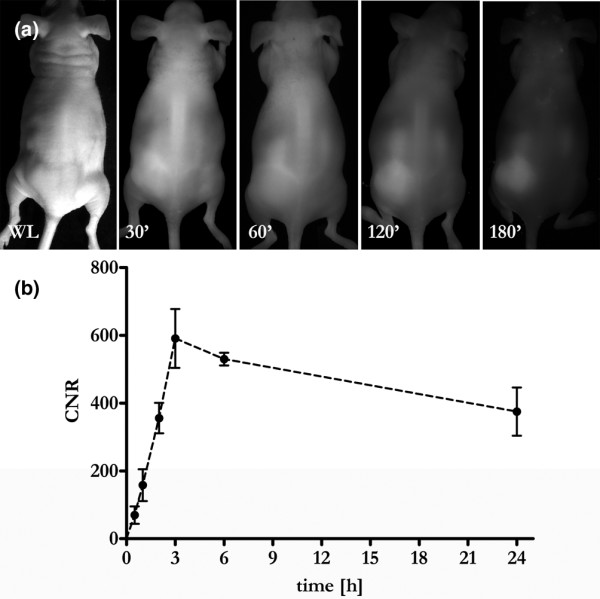
Contrast enhancement of a highly vascularized melanoma (MDA-MB435). **(a) **Fluorescence reflectance images were repetitively acquired up to 24 hours after injection of SIDAG (2 μmol/kg body weight). Note the strong enhancement of the tumour nodule, becoming increasingly conspicuous over the first 3 hours. Shown is a white light image, and fluorescence reflectance imaging images at 30, 60, 120 and 180 minutes after injection. **(b) **Quantitative data analysis revealed a rapid increase in the contrast to noise ratio (CNR) over the first 3 hours followed by a gradual wash out of the fluorochrome from the tumour tissue.

**Figure 3:**
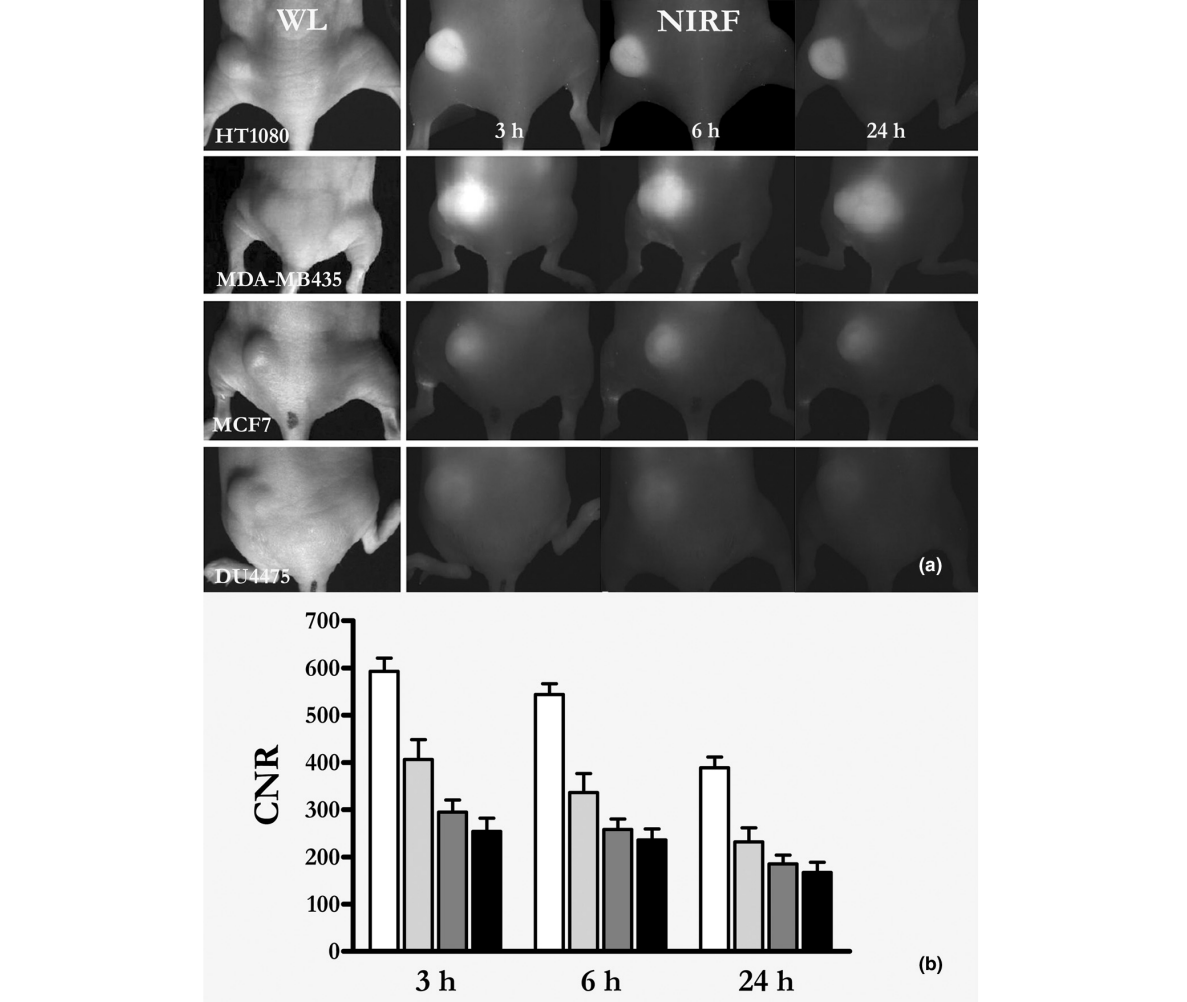
FRI of different tumour xenografts over time. **(a) **White light images of different tumour xenografts (as indicated), followed by fluorescence reflectance imaging (FRI) images taken sequentially after application of 2 μmol/kg body weight SIDAG. Note the strong fluorescence signal in the HT1080 (n = 11) fibrosarcomas and in the MDA-MB435 (n = 10) melanomas, whereas the MCF7 (n = 9) and DU4475 (n = 13) adenocarcinomas exhibit only moderate tumour fluorescence. **(b) **Quantitative data analysis revealed significantly higher contrast to noise ratios (CNRs) for the HT180 and MDA-MB435 xenografts compared with the MCF7 and the DU4475 tumours.

Sequential FRI during the first 90 minutes after intravenous administration of contrast agent yielded moderate CNRs in the tumour tissue, even in highly vascularized tumours such as MDA-MB435 xenografts (Figure 2). The tumours began to become discernible approximately 2 hours after injection, and maximal CNRs were observed 3 hours after injection. The fluorescence signal then slowly faded within the following 72 hours. Based on these results, the subsequent FRI and FMT experiments were performed 3, 6 and 24 hours after injection of SIDAG (Figure 3).

The results of all measurements are summarized in Table 1. In the FRI experiments, all xenografts were visible on FRI images at 3, 6 and 24 hours after injection. However, on comparing the different tumour types, MDA-MB435 melanomas exhibited the highest CNR at each time point, followed by the HT1080 fibrosarcomas, and the MCF7 and the DU4475 adenocarcinomas (*P *< 0.0001; Figure 3). Over time the CNR declined significantly while the signal differences among the various tumour types was unchanged (MDA-MB435 continued to exhibit the highest fluoresence signal intensity and DU4475 tumour xenograft the lowest; Figure 3).

**Table 1:** Summary of results for all tumour xenografts

Tumour model	CNR calculated by FRI (AU)^a^	FMT (nmol/l)	VVF (%)^b^	BVPD (BVPs/mm^2^)
MDA-MB435: melanoma	389 ± 72 (n = 10)	229 ± 90 (n = 4)	3.58 ± 0.9 (n = 4)	399 ± 36 (n = 6)
HT1080: fibrosarcoma	232 ± 99 (n = 11)	92 ± 65 (n = 5)	1.9 ± 0.7 (n = 4)	128 ± 38 (n = 6)
MCF7: adenocarcinoma	185 ± 57 (n = 9)	65 ± 50 (n = 6)	0.75 ± 0.5 (n = 4)	59 ± 10 (n = 6)
DU4475: adenocarcinoma	167 ± 79 (n = 13)	49 ± 22 (n = 4)	0.8 ± 0.5 (n = 4)	78 ± 16 (n = 6)

Data are expressed as mean ± standard deviation. Note that the data generated by fluorescence imaging technologies (FRI, FMT) correlate well with in in vivo (VVF) and ex vivo (BVDP) surrogate markers of tumour angiogenesis. ^a^Calculated 24 hours after intravenous injection of SIDAG. ^b^Measured using magnetic resonance imaging. AU, arbitrary units; BVP, blood vessel profile; BVPD: blood vessel profiles density; FMT, fluorescence-mediated tomography; FRI, fluorescence reflectance imaging; VVF, vascular volume fraction.

**Figure 4:**
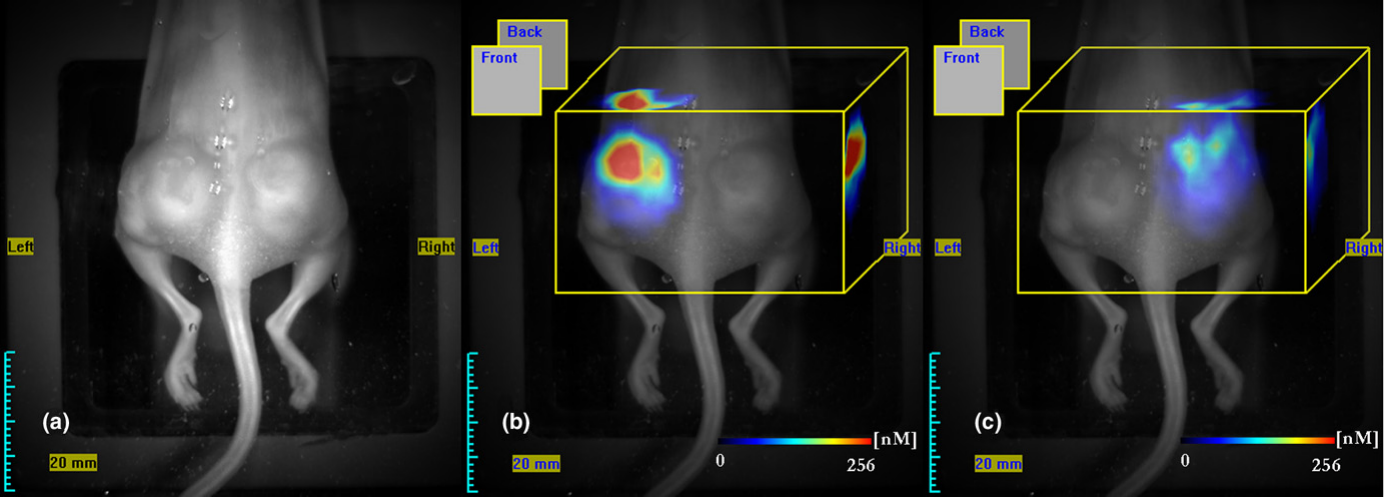
Quantification of fluorochrome accumulation in a double xenografted mouse by FMT. **(a-c) **Fluorescence-mediated tomography (FMT) was performed 24 hours after injection of SIDAG in order to resolve the three-dimensional fluorochrome distribution and for quantification of fluorochrome concentration in the target tissue: panel a shows a white light image, panel b shows colour-coded FMT data for MDA-MB435, and panel c shows the DU4475 xenograft superimposed on white light images. The average fluorochrome concentration in the MDA-MB xenografts ranged around 229 ± 90 nmol/l, whereas DU4475 exhibited significantly lesser fluorochrome accumulation (49 ± 22 nmol/l; *P *< 0.01).

Co-implantation experiments with mice bearing both the highly vascularized MDA-MB435 and poorly vascularized DU4475 tumours (n = 5) confirmed the previous FRI findings and allowed clear differentiation of MDA-MB435 melanomas from DU4475 adenocarcinomas within the same animal (Figure 4). Specifically, 3 hours after injection of SIDAG the CNR in the MDA-MB tumours was 553 ± 91 versus 195 ± 36 in DU4475 tumours (6 hours after injection: 508 ± 119 versus 167 ± 51; 24 hours after injection: 363 ± 84 versus 105 ± 20).

### Fluorescence mediated tomography

In order to assess the distribution profile and the concentration of SIDAG in deeper tissue sections more thoroughly, correlative FMT imaging was performed 24 hours after injection of the fluorochrome (Figure 4). FMT revealed an inhomogenous distribution of fluorochrome within the tumour volume (Figure 4). Double implanted tumour xenografts clearly differed in fluorochrome accumulation and were thus easily distinguishable by FRI and FMT (Figure 4). Maximum fluorochrome concentrations were found in the MDA-MB435 tumours (229 ± 90 nmol/l) whereas the fluorochrome accumulation in the other tumour types was significantly lower (HT1080: 92 ± 65 nmol/l; MCF7: 65 ± 50 nmol/l; DU4475: 49 ± 22 nmol/l; *P *< 0.05; Figure 4).

### Vascular volume fraction by magnetic resonance imaging

The independent *in vivo *MRI measurements showed an approximately 4.5-fold higher vascular volume fraction (VVF) in tumour tissue for MDA-MB435 (3.58 ± 0.9%) than for DU4475 (0.8 ± 0.53%; *P *< 0.01; Table 1). Likewise, the VVF for HT1080 (1.9 ± 0.67%; *P *< 0.05) and MCF7 (0.75 ± 0.52%; *P *< 0.01) were also significantly lower than for MDA-MB435 xenografts.

### Blood vessel profile density and VEGF expression

**Figure 5:**
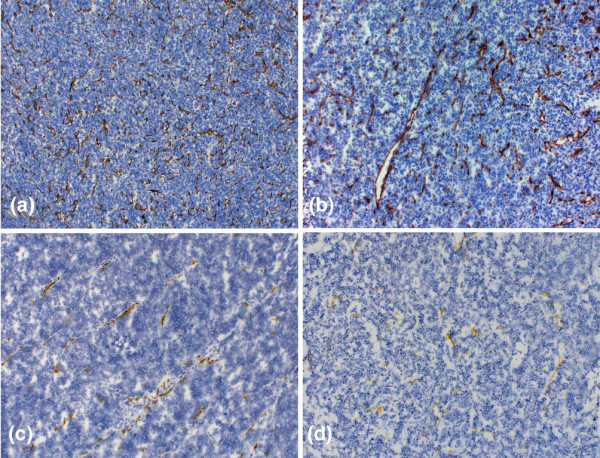
Immunhistochemistry of tumour xenografts. **(a-d) **Brown precipitates indicate the presence of CD31, representing microvessel formation: panel a shows MDA-MB435 tissue, panel b HT1080, panel c MCF7, and panel d DU4475. Note the strong difference in tumour vascularization. Although the MDA-MB435 and the HT1080 tumours exhibit a high degree of angiogenesis, the DU4475 and MCF7 tumours show a clearly lower amount of CD31 positive vessels (original magnification: 100×). Analysis of blood vessel profile density (BVPD) per area for all tumour types using stereological tools revealed the highest BVPD to be in MDA-MB435 (399 ± 36 BVPs/mm^2^) tumours followed by the HT1080 tumours (128 ± 38 BVPs/mm^2^), whereas the other two tumour types exhibited lower BVPD at a comparable level (MCF7 59 ± 10 BVPs/mm^2^; DU4475 78 ± 16 BVPs/mm^2^).

The density of the blood vessel profiles per area was highest in MDA-MB435 tumours (399 ± 36 BVPs/mm^2^), followed by the HT1080 tumours (128 ± 38 BVPs/mm^2^). A much lower BVPD was measured in the DU4475 (78 ± 16 BVPs/mm^2^) and MCF7 tumours (59 ± 10 BVPs/mm^2^; Figure 5 and Table 1). The highest expression of VEGF was found for MDA-MB435 tumour cells (100%), followed by HT1080 (90%), MCF7 (71%) and DU4475 (27%).

## Discussion

Optical imaging may provide ideal tools for noninvasive imaging of solid tumours such as breast cancer. Specifically, in the near infrared range, light can efficiently penetrate (breast) tissue, facilitating noninvasive probing of tissue with respect to oxyhaemoglobin and deoxyhaemoglobin, and fat and water content. Moreover, experimental data suggest that optical imaging is highly sensitive in resolving optical signatures, even in the picomolar range, which potentially can be exploited for molecular target detection *in vivo *[20].

Recent clinical data with nonenhanced diffuse optical tomography (computed tomography laser mammography) [5] demonstrated the basic feasibility of using nonenhanced optical tomography for breast cancer imaging. However, despite technological advances, including improved light generation, better detector systems and rigorous mathematical modelling, the findings of the present study corroborate previous reports [21-23] that found that nonenhanced optical mammography had limited sensitivity and specificity.

However, there is a good body of evidence that combining novel optical imaging technologies with fluorochrome-based optical contrast agents may markedly improve the sensitivity of the method, depending on fluorescent probe employed. In this regard, 'target-specific' or 'smart' fluorochromes can sensitively detect specific tumour epitopes, resolving structures even at picomolar concentrations, similar to scintigraphic imaging techniques.

Although target-specific and 'smart' fluorochromes can be applied to resolve molecular targets *in vivo*, the first clinical studies are currently being performed using non-target-specific, 'perfusion-type' fluorochromes, which are comparable to other clinically applied nonspecific contrast agents (for instance, gadolinum-based magnetic resonance or iodine-based radiographic contrast agents) [7]. It remains unclear whether these nonspecific fluorophores may be helpful in extrapolating relevant tissue parameters such as tissue perfusion, or whether they may even provide surrogate markers for tumour angiogenesis as an example.

The purpose of this study was therefore to apply SIDAG, a new generation, 'perfusion-type' fluorochrome, for two-dimensional and three-dimensional optical imaging in human cancer xenografts that significantly differ in their degree of tumour angiogenesis.

Our data reveal significant differences in tumour vascularization of the xenografts, as measured *in vivo *by iron oxide enhanced MRI as well as *ex vivo *by the BVPD and the level of VEGF expression as surrogate parameters of tumour angiogenesis. Planar and tomographic optical imaging revealed that retention of the fluorochrome in tumour xenografts made lesions clearly apparent. Interestingly, the retained fluorochrome concentration significantly increases with the degree of tumour angiogenesis, and both FMT and FRI data correlate with the VVF, BVPD and VEGF level. The data from this study therefore support the hypothesis that tumour-induced angiogenesis can indeed be noninvasively evaluated using a nonspecific, perfusion-type fluorochrome and appropriate imaging technology.

The first clinical feasibility study examining ICG-enhanced diffuse optical mammography was that presented by Ntziachristos and coworkers [7]. Those investigators examined patients with various breast lesions using ICG-enhanced diffuse optical mammography (off-label use) concurrently with contrast-enhanced MRI, and they demonstrated that it is indeed possible to resolve accumulation of ICG using diffuse optical tomographic methods and that ICG accumulation correlates with gadolinum-based signal enhancement in MRI [7].

The use of ICG in breast imaging is restricted by many factors. Because of a propensity to bind to albumin (95%), ICG distributes predominantly in the vascular space and undergoes rapid uptake by the liver. The plasma level of ICG falls within minutes after intravenous administration, which shortens the period during which imaging can be done [9]. Contrary to ICG, SIDAG has low plasma protein binding (10%) with the partition coefficient under 0.005 (ICG = ∞). Consequently, the SIDAG exhibits greater solubility and distributes in the extracellular space [24]. Further important features are greater fluorescent yield and tolerability.

In the present study the visibility of tumour xenografts with FRI increased substantially over the first 3 hours, showing a more than sixfold increase in CNR. This phenomenon can be attributed to the fact that SIDAG extravasated through leaky tumour vessels and was retained within the tumour interstitium over the first hours, whereas nontarget structures (nontumourous tissue) exhibited marked washout subsequently, which resulted in an increase in CNR.

The washin and washout phenomena described above are clearly depended on the tumour biology examined. Indeed, xenografts with a high degree of tumour angiogenesis (MDA-MB435 and HT1080) exhibited significantly higher SIDAG concentrations than did the poorly vascularized adenocarcinomas (MCF7 and DU4475).

Generally, factors such as vessel density, capillary permeability inside the tumours, or a combination of both may be responsible for greater enhancement of tumour tissue, because the microvascular hyperpermeability observed in carcinomas increases with increasing tumour grade [25]. This phenomenon is also well known from other imaging modalities such as gadolinum-enhanced MRI. Indeed, the degree of gadolinum extravasation and thus the signal increase is currently used as a diagnostic tool for the detection of breast cancer by MRI.

Compared with planar imaging technology (FRI), the FMT data more closely resembled the data generated by iron oxide enhanced MRI, which is a well established tool for the assessment of tumour angiogenesis. Although, for example, MRI revealed an approximately 4.5-fold higher VVF for MDA-MB435 than for DU4475 tumours, FRI revealed only a 2.3-fold higher fluorescence signal. However, FMT resolved an approximately 4.6-fold higher fluorochrome accumulation in the MDA-MB435 tumours as compared with DU4475 adenocarcinomas. This may be attributed to ability to resolve and differentiate deep-seated tissue fluorescence (such as that deriving from the liver) from more superficially located tumour-associated fluorochrome accumulation. Although FRI cannot differentiate between deep-seated strongly fluorescent structures and superficial fluorochrome accumulation, cross-sectional imaging with FMT can do so. Thus, scattered fluorescence from deep-seated structures can interfere significantly with imaging results with FRI as compared with FMT.

Contrary to most other clinically available cross-sectional imaging modalities, FRI and FMT allowed us to visualize the fluorochrome in highly vascularized tumours even 24 hours after injection with reasonable CNR. Moreover, the fluorochrome was administered at a dose of only 2 μmol/kg, which is more than two orders of magnitude lower than for gadolinum-enhanced MRI studies. These results therefore underline the high sensitivity of this imaging approach as compared with MRI, for example. More recent developments in the design of optical contrast agents include targeting tumour-specific receptors or the design of activatable 'smart' fluorochromes that can further boost the CNR, given an appropriate tracer design [26,27].

It is clear that fluorescence-enhanced optical imaging not only has tomographic applications in breast cancer imaging but also applications for a variety of other tumour types. Experimental studies have focused on imaging dysplastic colonic polyps, lung tumours and intracerebral malignomas [28]. Moreover, a combination with FRI applications in catheter-based or endoscopy-based imaging systems may be practical for a variety of other clinical scenarios [29]. Finally, real-time intraoperative FRI systems may greatly facilitate the detection of tumour margins and metastatic spread, such as to locoregional lymph nodes, and may therefore provide the surgeon with intraoperative guidance [30,31].

The present study has a few limitations: It remains unclear whether SIDAG-enhanced FMT/FRI can resolve subtle changes in vascular density or tumour perfusion, caused by antianiogenic therapy. Moreover, we did not conduct pharmacokinetic studies to analyze dynamically the distribution of the fluorochrome between intravascular and extravascular compartments. However, these issues will be addressed in future projects.

## Conclusion

The results of the present study indicate that clinically relevant tissue parameters such as tumour angiogenesis can reliably be estimated using a perfusion-type optical contrast agent and planar or tomographic optical imaging methods. These types of agents are about to enter the clincal arena, and their use in diffuse optical tomography of the breast may facilitate lesion detection and characterization.

## Abbreviations

BVP = blood vessel profile; BVPD = blood vessel profile density; CNBR = contrast-to-noise ratio; FMT = fluorescence mediated tomography; FRI = fluorescence reflectance imaging; MRI = magnetic resonance imaging; ICG = indocyanine green; ROI = region of interest; SIDAG = 1,1'-bis-(4-sulfobutyl)indotricarbocyanine-5,5'-dicarboxylic acid diglucamide monosodium; VEGF = vascular endothelial growth factor; VVF = vascular volume fraction.

## Competing interests

The authors declare that they have no competing interests.

## Authors' contributions

CB conceived the study, supervised the experiments and completed the final draft of the manuscript. AW and LM carried out the *in vivo *optical imaging experiments, analyzed the data and contributed to manuscript preparation. TP carried out the MRI experiments and participated in drafting the final manuscript. PH, KL, MS and SM developed the optical contrast agent, determined the tumour vessel density and contributed to drafting of the final manuscript. AvW carried out the western blotting. WH and AvW were involved in drafting the final manuscript.
